# The Role of Glucose, Serum, and Three-Dimensional Cell Culture on the Metabolism of Bone Marrow-Derived Mesenchymal Stem Cells

**DOI:** 10.4061/2011/429187

**Published:** 2011-04-05

**Authors:** Byron Deorosan, Eric A. Nauman

**Affiliations:** ^1^Weldon School of Biomedical Engineering, Purdue University, 585 Purdue Mall, West Lafayette, IN 47907-2088, USA; ^2^School of Mechanical Engineering, Purdue University, 585 Purdue Mall, West Lafayette, IN 47907-2088, USA; ^3^Department of Basic Medical Sciences, Purdue University, 585 Purdue Mall, West Lafayette, IN 47907-2088, USA

## Abstract

Mesenchymal stem cells (MSCs) have become a critical addition to all facets of tissue engineering. While most *in vitro* research has focused on their behavior in two-dimensional culture, relatively little is known about the cells' behavior in three-dimensional culture, especially with regard to their metabolic state. To evaluate MSC metabolism during twodimensional culture, murine bone marrow-derived MSCs were cultured for one week using twelve different medium compositions, varying in both glucose and fetal bovine serum (FBS) concentrations. The results indicate that glucose concentration was the more important factor in sustaining cell growth and viability. To evaluate metabolic state during three-dimensional culture, MSCs were cultured for one week using two different medium compositions and two different concentrations of collagen gel matrix. The medium compositions only varied in glucose concentration. The results indicate that glucose and extracellular matrix were significant factors in the metabolic response of the cells. However, cells cultured in low density collagen exhibited considerable cell death, likely because of physical contraction of the collagen hydrogel which was not observed in the higher density collagen. These findings will be useful to the development of *in vitro* cell culture models that properly mimic *in vivo* physiological processes.

## 1. Introduction

Tissue engineering integrates the application of engineering and biological principles to study, design, develop, and repair biological structures. It is an iterative, objective-driven process which has spurred the production of artificial skin [1–3] and led to advances in the development of smooth muscle tissue [4, 5], synthetic blood vessels [6–8], cardiac tissue analogues [9–11], renal tubules [12, 13], intestinal segments [14, 15], bladder substitutes [16–18], and bone tissue scaffolds [19–21]. Long-term success of these tissue-engineered therapies requires their biocompatibility with the host tissue and the development of functionally differentiated cells within the implanted tissue. Stem cells are important to these applications because of their ability to differentiate into various cell types. 

Bone marrow-derived mesenchymal stem cells (MSCs) are less controversial to obtain and easier to control than embryonic stem cells [22]. But, all stem cells must be subjected to various environmental cues in order to differentiate into specific cell types. To date, the most common method of controlling stem cell differentiation is to implement physiologically relevant factor proteins and molecular cues designed to take advantage of the natural competence of the MSCs. This process has led to advances in liver repair [23–28], islet cell regeneration [29–34], bone augmentation [35–37], and spinal cord regeneration [38–40]. 

While these advances are significant to the field, current research is lacking in relation to the three-dimensional culture of MSCs, their metabolic state, and how both of these considerations may affect their terminal differentiation. It is important to consider how cells will respond in three-dimensional culture compared with two-dimensional culture because it has been shown that extracellular matrix interactions cause many different cellular reactions related to differentiation, proliferation, growth, motility, and gene expression [41–49]. Additionally, because aerobic metabolism and anaerobic metabolism are the primary means of deriving energy for cells and because much more energy is derived aerobically than anaerobically, it is reasonable to assume that the prevailing metabolic state experienced by the cells will have a significant effect on the terminal differentiation of the stem cells. Evidence has shown that metabolic state can influence both protein activation and protein conformation [50, 51], both of which are crucial during cell differentiation. This may also affect the ability of growth factors and other stimuli to induce the desired outcomes in vivo.

Consequently, the overall motivation of this series of experiments was to investigate the metabolic state of MSCs in culture in response to variations in glucose and fetal bovine serum (FBS) concentrations in both two-dimensional and three-dimensional culture conditions.

## 2. Materials and Methods

### 2.1. Two-Dimensional Cell Culture

Murine MSCs (ATCC Number CRL-12424) were used for these experiments (ATCC, Manassas, VA, USA). MSCs were cultured in maintenance medium composed of glutamine-free Dulbecco's modification of Eagle's medium (DMEM) (90-113-PB, Mediatech, Herndon, VA) supplemented with 10% FBS (Invitrogen, Carlsbad, Calif, USA), 1% penicillin-streptomycin (10 mg/mL, Sigma-Aldrich, St. Louis, Mo, USA), and 0.1% amphotericin-B (250 *μ*g/mL, Sigma-Aldrich, St. Louis, MO). The glucose concentration within this medium was made to be 25.0 mM by adding D-glucose (4912, Mallinckrodt, Phillipsburg, NJ, USA). MSCs were seeded in either 6-well or 24-well plates (BD Biosciences, San Jose, Calif, USA) at a density of 1.5×10^4^/cm^2^. The 6-well plates were used to measure samples for the metabolic assays, and the 24-well plates were used to acquire cell number at various days throughout the experiment. At this density, the cells were nearly confluent upon seeding, in order to minimize cell proliferation over the course of the experiment. Upon seeding the cells, wells in the 6-well and 24-well plates were filled with 2.4 mL and 0.5 mL of maintenance medium respectively so that the medium height would be the same between the two plate types. The cells were allowed to attach and stabilize for twelve hours after seeding. The immediate conclusion of this twelve-hour period corresponds to “day 0.” The study lasted a total of six days. Twelve experimental medium groups were used in this portion of the study corresponding to twelve glucose concentration (0.5 mM, 1.0 mM, 5.0 mM, and 25.0 mM) and serum concentration (2%, 5%, or 10%) combinations. Twelve samples were accorded to each group, except the pyruvate groups (please see below). Subsequently, 1.0 mL medium samples were obtained on days 4 and 6 for the glucose, lactate, and pyruvate analysis. Cell number was measured on days 0, 2, 4, and 6. Medium was refreshed in wells that were not analyzed on that particular experimental day.

### 2.2. Three-Dimensional Cell Culture

Circular collagen gels with an area of 2.0 cm^2^ and a height of 0.5 mm were used as three-dimensional scaffolds for culturing the MSCs. Collagen was harvested from porcine tendon and placed in 0.5 M acetic acid (Mallinckrodt Baker, Phillipsburg, NJ). The acid soluble collagen was neutralized with 2.0 M sodium hydroxide (NaOH) (Mallinkrodt Baker, Phillipsburg, NJ). High-density gels at 5.0 mg/mL and low-density gels at 2.5 mg/mL were prepared in 24-well plates. Each gel was formed using 100 *μ*L of collagen solution and each contained 250,000 MSCs. Four experimental groups were used in this portion of the study corresponding to two glucose concentration (0.5 mM and 25.0 mM) and final collagen concentration (2.5 mg/mL and 5.0 mg/mL) combinations. Twelve samples were accorded to each group. The serum concentration for each case was 5%. For each medium combination, a volume of 0.5 mL was used for each well of the 24-well plates. Subsequently, the medium was collected on days 4 and 6 for the glucose and lactate characterization. Cell number was measured on days 0, 2, 4, and 6.

### 2.3. Cell Population

The 24-well plates were used for cell number determination for both two- and three-dimensional cultures. Briefly, Hoechst dye (Invitrogen, Carlsbad, Calif, USA) was added to DMEM for a final concentration of 5.0 *μ*g/mL. A 100 *μ*L volume of this Hoechst solution was added to each well of the 24-well plates. Subsequently, the plates were placed in a 37°C, 5% CO_2_ incubator for fifteen minutes. Each plate was read in a fluorometer (Thermo Electron, Finland) using an excitation of 355 nm and the emission was measured at 460 nm. Background measurements were taken using cell-free empty wells for the two-dimensional culture or cell-free gels for the three-dimensional culture. The fluorometer readings, in conjunction with an experimentally determined calibration curve, were used to calculate the cell population within each well. 

### 2.4. Cell Viability

The same 24-well plates used to assess cell population were used to observe cell viability qualitatively using fluorescence imaging. Briefly, both calcein-AM and ethidium bromide (Fluka, St. Louis, MO) were added to DMEM for a final concentration of 2.5 *μ*M. Calcein-AM indicates living cells by emitting 517 nm light when excited by 494 nm light, and ethidium bromide indicates dead cells by emitting 617 nm light when excited by 528 nm light. A 100 *μ*L volume of this solution was added to each well of the 24-well plates. Subsequently, the plates were placed in a 37°C, 5% CO_2_ incubator for fifteen minutes. The wells were observed using the 10X and 40X magnification objectives on an IX71 fluorescence microscope (Olympus, Center Valley, Pa, USA), and TIFF images were taken using a QColor 5 camera (Olympus, Center Valley, PA) and QCapture Pro version 5.0.1.26 software (QImaging, Surrey, BC, Canada). The TIFF images were converted to bitmap images using Irfanview version 4.25 software (Irfan Skiljan, 2009) and were then merged using Artweaver version 0.3.9.9 software (Boris Eyrich, 2005).

### 2.5. Glucose Assay

Medium samples were tested for glucose concentration using a fluorometric assay kit (Invitrogen, Carlsbad, CA). The kit indicates the presence of glucose by activating fluorescent resorufin, which has absorption maxima at 571 nm and emission maxima at 585 nm. Samples were diluted with deionized water so that the glucose concentration would fall within the optimum detection range of the kit, between 3.0 *μ*M and 50.0 *μ*M. Fluorescence was measured using a fluorometer with excitation at 530 nm and emission detection at 590 nm. Background measurements were taken using glucose-free DMEM (Invitrogen, Carlsbad, CA). The emission values were converted to glucose concentration using a calibration curve. Then, these concentrations were multiplied by the dilution factor to obtain the glucose concentration within each extracellular sample. This resulting concentration was subtracted from the actual starting glucose concentration of the original growth medium to obtain the glucose consumption.

### 2.6. Lactate Assay

Medium samples were tested for lactate concentration using a spectrophotometric assay kit (Instruchemie, The Netherlands). The kit determines the concentration of lactate by detecting an increase in the absorbance of reduced nicotinamide adenine dinucleotide (NAD) at 340 nm, indicating the amount of lactate originally present in the sample. The NAD absorbance at 340 nm was measured with a spectrophotometer (Thermo Electron, Finland). Extracellular samples were diluted with an equal amount of deionized water so that the lactate concentration would fall within the optimum detection range of the kit, between 0.22 mM and 13.32 mM. Background measurements for extracellular samples were taken using ordinary DMEM. These background measurements were subtracted from their respective absorption measurements. Background-subtracted measurements were converted to lactate concentrations using a calibration curve.

### 2.7. Pyruvate Assay

Extracellular and intracellular samples were tested for pyruvate concentration using a spectrophotometric assay kit (Instruchemie, The Netherlands). The kit determines the concentration of pyruvate by detecting a decrease in the absorbance of oxidized nicotinamide adenine dinucleotide** (**NAD) at 340 nm, indicating the amount of pyruvate originally present in the sample. The NADH absorbance at 340 nm was measured with a spectrophotometer (Thermo Electron, Finland). The optimum detection range of the kit was between 0.011 mM and 0.340 mM. 

Pyruvate measurements were taken using both an intracellular and extracellular approach. The extracellular approach was performed in both the two- and three-dimensional conditions with six samples accorded to each condition. The extracellular approach consisted of measuring the pyruvate concentration within the same medium samples used for glucose and lactate measurements. The intracellular approach was only performed for the two-dimensional case. Briefly, each well of the 6-well plate was rinsed twice with PBS after the medium sample was extracted for extracellular measurements. Next, the cells were removed from the wells using cell scrapers, 0.5 mL of cold perchloric acid was added to each well, and the cell/acid solution was placed in microcentrifuge tubes. Next, the samples were subjected to vortex for thirty seconds, placed in a −20°C freezer for five minutes, and then centrifuged for ten minutes at 1700 ×g (Beckman Coulter, Fullerton, CA). The resulting supernatant was used to assay for pyruvate concentration. The samples did not have to be diluted in either the extracellular or intracellular case. Background measurements for intracellular samples were taken using deionized water samples. Background measurements for extracellular samples were taken using ordinary DMEM. These background measurements were subtracted from their respective absorption measurements. Background-subtracted measurements were converted to lactate concentrations using a calibration curve.

### 2.8. Characterization of the Gel Transport Properties

Viscous drag coefficients were obtained for trypan blue (Sigma-Aldrich, St. Louis, MO) diffusing through 2.5 mg/mL and 5.0 mg/mL collagen gels. Clear 1.0 cm diameter test tubes were used to hold 2.0 mL of each gel concentration (*N* = 3 for each concentration). The tubes were then capped and placed in a 37°C incubator for two hours. After incubation, each received 1.0 mL of 0.13% trypan blue solution. The trypan blue solution was allowed to diffuse through the collagen gels while the tubes were maintained at 37°C. Over the span of 3 days, the tubes were periodically photographed (Canon EOS Rebel Digital EF-S 18-55; Tokyo, Japan) and the images were used to determine the distance that the trypan blue solution advanced through the gels using QCapture Pro software (version 5.0.1.26; QImaging; Surrey, BC, Canada). The process was repeated for a set of trypan blue solutions in pure water. After all of the data was collected, the trypan blue advancement was plotted versus time. A second-order polynomial curve was fit to the data points, and the equation was used to find values for both the velocity and acceleration of the dye through the gels. A force balance on the trypan blue molecules was used to estimate values of the drag coefficient

(1) $$-\zeta *v_{\text{drift}}-\rho_{\text{gel}}Vg+\rho_{\text{trypan}}Vg=\rho_{\text{trypan}}Va,$$

where **ζ** is the drag coefficient, *v*_drift_ is the drift velocity of the trypan blue molecules, *ρ*_gel_ is the density of the gel, *V* is the volume of the molecule that is diffusing through the matrix, *g* is the acceleration due to gravity, *ρ*_trypan_ is the density of trypan blue powder, and *a* is the experimentally determined acceleration of the trypan blue through the collagen matrix. For these experiments, the values of the constants in (1) were the following: *V* was 1.20*E* − 28 m^3^, *ρ*_trypan_ was 1667 kg/m^3^, *ρ*_gel_ was 1000 kg/m^3^, and *g* was 9.8 m/s^2^. The same values were used to estimate the drag coefficient for trypan blue in water. Afterward, (1) was used to solve for relative drag coefficients consisting of the drag coefficients of trypan blue in collagen gels normalized to that of trypan blue in water. It is critical to note that experimental values for the acceleration of the trypan blue molecule were much smaller than those of the acceleration due to gravity. Therefore, the term containing *a* was negligible in solving for the relative drag coefficients. Relative diffusivity values were calculated by taking the reciprocal of the respective drag coefficients.

### 2.9. Statistics

StatView version 5.0.1 was used to apply a one factor analysis of variance (ANOVA) and Tukey-Kramer post-hoc test to the data to evaluate statistical significance between the groups at a significance level of *P* < .05.

## 3. Results

### 3.1. Cell Population

**Figure 1:**
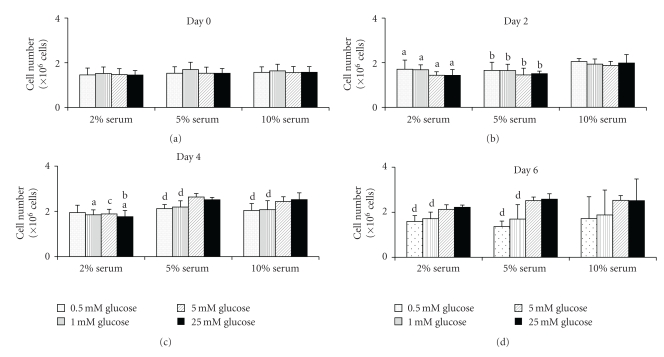
Cell population (mean ± standard deviation) over the course of six days while in two-dimensional culture (*n* = 12 per group). Higher glucose and serum concentrations appear to produce higher cell populations over time. (a: significance between corresponding value in the 5% group; b: significance between corresponding value in the 10% group; c: significance between corresponding values in the 5% and 10% groups; d: significantly difference between the 5.0 and 25.0 mM glucose values within the same serum group; *P* = .05).

**Figure 2:**
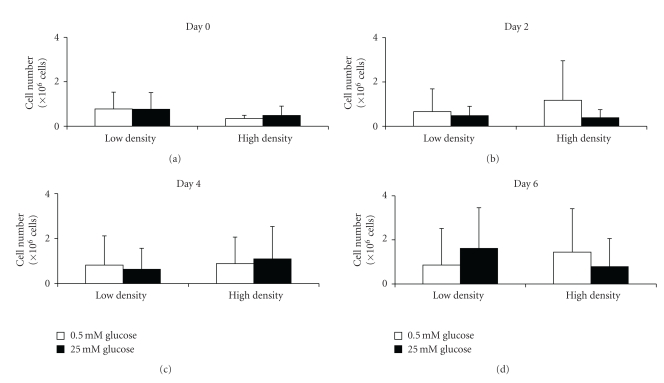
Cell population (mean ± standard deviation) over the course of six days while in three-dimensional culture (*n* = 12 per group). Culturing the cells within the differing three-dimensional environments did not produce any particular trend in the cell population. Statistical significance was not observed at the *P* = .05 level.

The cell population at the start of the two-dimensional experiment was even between the different experimental groups (Figure 1). The population data began to exhibit significant differences between serum concentrations at day two and between glucose concentrations at day four. However, by day six significant differences between serum groups were not found. Additionally, higher glucose concentrations produced higher cell populations than the lower glucose concentrations. The three-dimensional cell population data did not exhibit significant differences between the two collagen densities or the two glucose concentrations (Figure 2). However, of note are the high standard deviations for most of the data.

### 3.2. Cell Viability

**Figure 3:**
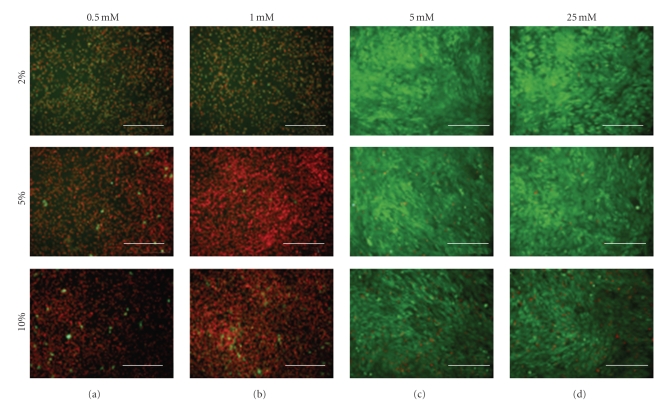
Fluorescence microscopy images indicating the viability of BMSCs in two-dimensional culture after the second day (increasing glucose concentrations from left to right; increasing serum concentrations from top to bottom). Calcein-AM indicates living cells by emitting green light, while ethidium bromide indicates dead cells by emitting red light. The 0.5 mM and 1.0 mM environments resulted in low viability and sparse cell distribution. The 5.0 mM and 25.0 mM environments resulted in high viability and the maintenance of high confluency. Varying the serum concentration did not produce a noticeable difference in the viability. This result was consistent throughout the course of the week (20x objective; scale bar represents 250 *μ*m).

**Figure 4:**
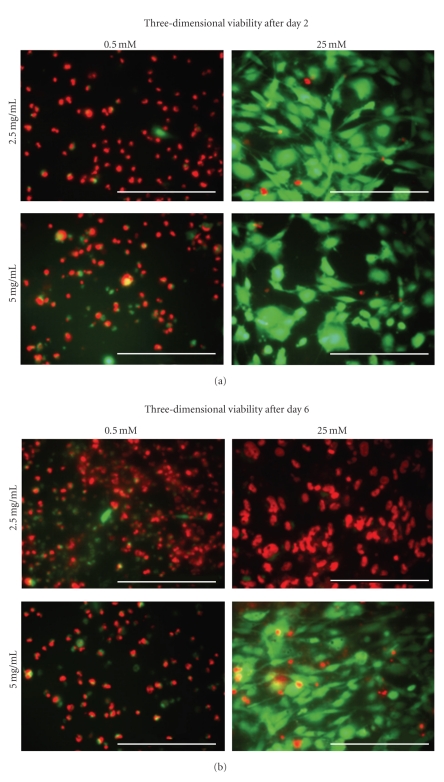
Fluorescence microscopy images indicating the viability of BMSCs in three-dimensional culture after the second and sixth days (increasing glucose concentrations from left to right; increasing collagen densities from top to bottom). Calcein-AM indicates living cells by emitting green light, while ethidium bromide indicates dead cells by emitting red light. After day 2, the 0.5 mM environment resulted in low viability, while the 25.0 mM environment resulted in high viability. By day 6, the 0.5 mM environment and the 25.0 mM−2.5 mg/mL group resulted in low viability, while the 25.0 mM−5.0 mg/mL group resulted in high viability and confluency. (40x objective; scale bar represents 100 *μ*m).

The two-dimensional, day zero cell viability indicates high cell number and confluency before the cells were exposed to any experimental treatments (images not shown). However, by day two the cells in the 0.5 mM and 1.0 mM environments exhibited low viability while those in the 5.0 mM and 25.0 mM environments exhibited high viability (Figure 3). This trend continued through days four and six. For the three-dimensional culture, there were noticeable differences in the cell viability between both the collagen densities and the environmental glucose concentrations. At day zero, the cell viability and confluency were higher for cells placed in the 2.5 mg/mL collagen matrix (images not shown). Once applied, the 0.5 mM glucose environment caused the cells to exhibit low viability and confluency for the duration of the experiment regardless of the collagen density (Figure 4). In the 25.0 mM glucose group, cell viability and confluency steadily decreased in the 2.5 mg/mL group while viability and confluency increased in the 5.0 mg/mL group (Figure 4).

### 3.3. Glucose Consumption

In both the two- and three-dimensional cases, glucose consumption was the highest in the 25.0 mM groups (data not shown). For the two-dimensional case, there were three cases of significant differences between serum groups and a number of significant differences between glucose groups. The three-dimensional case did not yield a significant difference between collagen groups or glucose groups until day six, when the consumption was higher for the cells in the 5.0 mg/mL collagen matrix than the cells in the 2.5 mg/mL collagen matrix.

### 3.4. Lactate Production

Groups with higher glucose consumption would be expected to yield higher lactate production as the endpoint of glycolysis. This behavior was shown in the lactate production data, with lactate production increasing with increasing glucose consumption in both two- and three-dimensional cultures (data not shown). At all FBS levels on days 4 and 6 the 0.5 mM and 1.0 mM glucose groups exhibited significantly lower lactate production than the 15 mM group at the *P* < .05 level. Similarly, lactate production mirrored the glucose consumption in three-dimensional culture for both days four and six. There was no significant effect of FBS on lactate production in the two dimensional cultures.

### 3.5. Pyruvate Production

**Figure 5:**
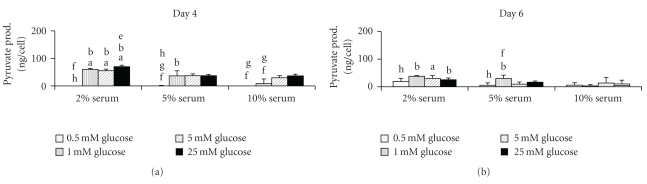
Extracellular pyruvate production after the final two days of two-dimensional culture (*n* = 12 per group). The production data does not show a clear trend. (a: significantly different from corresponding value in the 5% group; b: significantly different from corresponding value in the 10% group; e: significantly different from all other values within same serum group; f: significantly different from 5.0 mM value within same serum group; g: significantly different from 25.0 mM value within same serum group; h: significantly different from the 1.0 mM value within the same serum group; *P* = .05).

As pyruvate is not likely to accumulate, being converted to either lactate or acetyl coenzyme A, the amount of pyruvate produced would not be expected to have a consistent pattern amongst the experimental glucose groups. This random behavior was shown in both the intracellular and extracellular, two-dimensional culture data, especially in terms of statistical significance (Figure 5). However, the overall pyruvate levels did decrease as the serum concentration increased in both of the two-dimensional cases. The pyruvate production levels in the three-dimensional culture were consistently higher for the 25.0 mM glucose group than the 0.5 mM glucose group.

### 3.6. Metabolic Ratios

Lactate-to-pyruvate ratios for two-dimensional culture showed an increasing trend with an increase in the starting glucose concentration, indicating that higher glucose cultures were more anaerobic (Table 1). For the lactate-to-glucose ratios for two-dimensional culture, the values within the 2% serum group exhibited a similar increasing trend with an increase in starting glucose concentration (Table 2). However, the values within the 5% and 10% serum groups did not. Of note are the discrepancies in the actual sample populations when evaluating statistical significance. Many samples showed little to no pyruvate production, causing calculation of the lactate-to-pyruvate ratios to become impossible. Additionally, some samples showed little to no glucose consumption in the 0.5 and 1.0 mM groups, causing lactate-to-glucose ratio calculations to become impossible as well. This explains why a number of experimental groups lack the expected twelve samples. The lactate-to-pyruvate and lactate-to-glucose ratios for the three-dimensional culture did not show any clear trend (Table 2).

### 3.7. Gel Transport Properties

Drift velocities and accelerations indicate that the trypan blue molecules traveled quicker through the 2.5 mg/mL gel than the 5.0 mg/mL gel (Table 3). These characteristics were reflected in the mean drag coefficients, as that of the 5.0 mg/mL gel was larger than that of the 2.5 mg/mL gel.

## 4. Discussion

The overall purpose of this research was to investigate possible differences in the metabolic state of MSCs when cultured in ordinary two-dimensional culture versus collagen-based three-dimensional culture. The two-dimensional experiments consisted of subjecting the cells to various medium types that differed in both glucose and serum concentrations. The three-dimensional experiments subjected cells to two different glucose concentrations and two collagen densities. The three-dimensional cultures were only subjected to variation in glucose concentration as glucose appeared to have the most critical effect on cell growth, viability, and metabolic state. The 5% serum concentration was chosen because of its moderation between the 2% and 10% serum groups. The results highlight relative differences in the metabolic state of MSCs when cultured within these differing conditions. Overall, the two most important factors in affecting the cell population were starting glucose concentrations and the addition of a three-dimensional matrix. 

This result could have important implications for stem cell therapies, as differences in metabolic state during culture may affect the terminal differentiation of the MSCs. Human adipose-derived stem cells have already shown evidence of this as their differentiation into osteogenic cells is altered by changing their metabolic conditions [52]. As differentiation is a complex process undertaken by stem cells, it is reasonable to assume that achieving any specific terminal differentiation is dependent upon a significant amount of energy use. Both aerobic metabolism and anaerobic cell metabolism produce the energy that all cells depend upon to carry out physiological processes. Furthermore, evidence has shown that cells exhibit a preference for one metabolic state over the other for specific processes [50, 51]. 

Anaerobic glycolysis and the aerobic citric acid cycle are the two primary means for cells to obtain energy. They can be distinguished by the fate of the glucose consumed by the cells. In glycolysis, the glucose is converted to pyruvate, which is then converted to lactate as a dead-end product. However, in the citric acid cycle, the pyruvate produced at the conclusion of glycolysis enters into the citric acid cycle in the form of acetyl coenzyme-A. The relative metabolic state—whether aerobic or anaerobic—of a cell culture can be assessed after measuring the glucose consumed and lactate produced; the higher the amount of lactate, the more likely that the relative metabolic state anaerobic. 

These data elucidate the ability of growth medium glucose and serum concentrations to alter the metabolic state of the MSCs. It has already been shown that adipose-derived stem cells from rabbits [53] and humans [52] increase their lactate production, proliferation, and viability when exposed to higher glucose environments. The results of this series of experiments correspond well with those outcomes. The metabolic ratios for the two-dimensional experiments indicate that both the serum and glucose concentrations within the growth medium help to determine the cell's metabolic state, with higher serum concentrations and higher glucose concentrations leading to more anaerobic cultures. The lactate-to-pyruvate ratios indicate that glucose was more influential than serum, as increases in the glucose concentration produced marked increases in the metabolic ratio that were not replicated by increases in the serum concentration. The ratios for the three-dimensional experiments did not show a clear trend across the last two days. But overall, the lactate-to-pyruvate ratios were much lower for the three-dimensional cultures than for the two-dimensional cultures, indicating that the three-dimensional cultures were less anaerobic. While the results of the two dimensional cultures were expected, the addition of a three dimensional extracellular matrix had unexpected effects on the L/P and L/G ratios, suggesting that these effects must be investigated in much more detail. 

The setup of these experiments had several advantages. The primary advantage was the use of the same cell type for both two-and three-dimensional culture so as to avoid potential significant differences in metabolism between different cell types. This reality was highlighted by Sander and Nauman, as it was found that culturing rat astrocytes produced lower lactate-to-glucose ratios than both porcine lamina cribrosa cells and porcine scleral fibroblasts after one week of culture in environments with different glucose and oxygen levels [54]. Additionally, in the case of the two-dimensional experiment, both glucose and serum concentrations were varied simultaneously so that the effects of both medium components could be isolated. The glucose assay, with a resolution of 3.0 *μ*M of D-glucose, the lactate assay, with a resolution of 0.22 mM of lactate, and the pyruvate assay, with a resolution of 0.011 mM, provided high sensitivity. In the future, studies of this nature will be supplemented with real-time imaging of NADH within live cells to estimate the location and intensity of the relative metabolic activity within the cells.

These experiments also possessed a number of limitations. The purpose of the trypan blue diffusion experiments was to investigate the possibility that the metabolites were trapped within the collagen matrix, thus avoiding detection by the assays. However, trypan blue—molecular weight of ~960 g/mol—was able to seep approximately 1.0 mm through both the 2.5 mg/mL and 5.0 mg/mL collagen gels in about fifteen minutes (data not shown). Therefore, it is reasonable to assume that glucose, lactate, and pyruvate—all with molecular weights of less than 200 g/mol—were able to traverse the collagen matrix and be subject to detection. There are two other circumstances that indicate that the thickness of the gels did not prevent adequate transport of lactate out of the gels. The first is the recognition that calcein-AM—molecular weight ~995 g/mol—did not have any trouble diffusing into the gel within fifteen minutes in order to take fluorescence images of the cells. The second comes from the result of the nutrient supply study by Horner and Urban [55]. In this study, the researchers embedded nucleus cells within a 1% agarose matrix and monitored the cells' viability as a function of their distance away from the nutrient supply within a diffusion chamber. Homer and Urban showed that the embedded cells were still viable within 5.0 mm of the nutrient supply. However, there is uncertainty as to the similarity of diffusive and mechanical properties between the 1% agarose gels and the collagen gels of this study. Another disadvantage was that constriction of the collagen gels could not be prevented during the course of the week long experiments, with most of the gels constricting to about 75% of their original size. This may especially be reflected in the cell viability images, in which higher cell death is seen in the 2.5 mg/mL samples rather than the 5.0 mg/mL samples by the time the experiment concluded. It is likely that the stiffer gels attenuated the degree of constriction, giving the cells better access to nutrient/waste transport through the matrix during the course of the week.

The results of this series of experiments serve as a baseline characterization of MSCs in culture before being subjected to either an extracellular matrix or a differentiation scheme, and they will be especially beneficial to the tissue engineering field. While it is well documented that extracellular matrices have significant effects on several cell types [56–59], the effect of extracellular matrices on cell metabolism have yet to be investigated. Because cell metabolism is altered in three-dimensional culture when compared to two-dimensional culture, this could have significant implications on the use of extracellular matrices because cell metabolism affects all of cellular physiology. Therefore, a future study on the effect of cell metabolism on stem cell terminal differentiation is imperative. Additionally, it will be equally important to perform a study investigating differences in cell metabolism versus the type of extracellular matrix material used. Herein, we demonstrated that glucose is the dominant factor affecting cell metabolism, but FBS also modulates this effect, and the long-term effects of culture conditions on phenotype and phenotypic potential must still be elucidated. It is anticipated that FBS availability may play a more important role in differentiation, and it is possible that the protein content of the FBS could play a role in coupling the metabolic and differentiation pathways. 

While, there is always great risk associated with transitioning from in vitro to in vivo studies because the environments are different in many ways, from practical and ethical standpoints, it would be advantageous to have a means of investigating a set of experimental variables on an artificial tissue analogue prior to implementing the same variables in an animal system; this intermediate step could accelerate research findings and prevent the unnecessary deaths of the animals should the results from this in vitro tissue reveal dangerous effects that the two-dimensional case alone did not reveal. Consequently, this intermediate step could potentially decrease costs associated with in vitro-to-in vivo experimentation.

## 5. Conclusions

The results of this study indicate that the amount of glucose provided and the presence of an extracellular matrix do indeed affect the metabolic state and viability of cells in culture. Providing larger concentrations of glucose may be a way to push the cells toward the anaerobic state, all else being equal. Additionally, the results demonstrated that the three-dimensional culture of MSCs may have to take place within a relatively stiff matrix or a composite of collagen hydrogel and collagen fibers [60, 61] to prevent decreases in cell viability, and further work is needed to elucidate the effects of extracellular matrix on MSC metabolism. Diffusion results show that the overall transport properties of two high-density collagen gels were essentially the same, so that differences in the results between experimental groups in three-dimensional culture could not be attributed to differences in transport properties. Further investigation is required to elucidate the effects of metabolic state on phenotype and phenotypic potential.

## Figures and Tables

**Table:** (a) Two-dimensional lactate-phosphate (L/P) ratios.

	Glucose conc. 2% serum				5% serum			10% serum		
		Mean	St. Dev.	*N *	Mean	St. Dev.	*N *	Mean	St. Dev.	*N*
Day 4	0.5 mM	2.41	2.36	3	2.85	2.92	6	5.92	3.48	3
	1.0 mM	0.59^b^	0.93	9	4.77^b^	4.18	10	5.61	3.02	7
	5.0 mM	8.79	8.52	12	23.46	17.01	10	52.48	34.08	8
	25.0 mM	14.98	13.12	12	36.05	34.25	12	50.01	32.62	11

Day 6	0.5 mM	0.17	0.34	8	9.57	17.89	4	4.15	4.83	4
	1.0 mM	10.06	25.28	11	26.58	11.34	6	31.76	37.43	4
	5.0 mM	51.37	17.22	6	134.35	111.21	8	234.68	549.5	10
	25.0 mM	71.49	59.71	10	113.18	76.5	12	114.8	152.36	9

**Table:** (b) Two-dimensional lactate-glucose (L/G) ratios.

	Glucose conc. 2% serum				5% serum			10% serum		
		Mean	St. Dev.	*N *	Mean	St. Dev.	*N *	Mean	St. Dev.	*N*
Day 4	0.5 mM	0.72^a^	0.45	8	1.47	0.09	6	0.57	0.62	12
	1.0 mM	0.52	0.55	12	1.81^b^	1.44	12	2.71	3.65	10
	5.0 mM	1.19	0.89	12	2.2	1.42	12	2.09	1.44	12
	25.0 mM	1.53	1.56	12	1.9	1.49	12	2.85	2.67	12

Day 6	0.5 mM	0.65^f^	0.49	9	1.94	2.52	8	0.59	0.63	12
	1.0 mM	0.47^f^	0.49	12	3.65	2.6	12	13.53	22.57	9
	5.0 mM	2.39	1.75	12	2.99	2.29	12	2.69	2.07	12
	25.0 mM	1.56	1.2	12	1.95	1.45	12	2.32	1.91	12

**Table 2:** Summary of three-dimensional metabolic ratios. There was no clear trend in the data. (a: significantly different from corresponding value in the 5.0 mg/mL group; b: significantly different from the 25.0 mM value within the same collagen group; *P* = .05).

	Experimental Group L/P Ratio				L/G Ratio		
		Mean	St. Dev.	*N *	Mean	St. Dev.	*N*
Day 4	0.5 mM/low density	2.30^b^	5.66	12	1.93	2.29	6
	25.0 mM/low density	11.92^a^	5.41	7	0.92^a^	0.97	12
	0.5 mM/high density	2.15^b^	2.42	12	15.47^b^	11.20	8
	25.0 mM/high density	18.73	4.95	6	1.11	0.88	12

Day 6	0.5 mM/low density	74.75^a,b^	77.85	3	17.30^a^	47.92	12
	25.0 mM/Low Density	8.49	9.85	12	2.34	2.44	8
	0.5 mM/high density	2.92	1.15	6	11.04^b^	8.60	8
	25.0 mM/high density	6.32	7.23	12	1.02	1.22	12

**Table 3:** Summary of results pertaining to trypan blue diffusion through collagen gels. The diffusivity for both cell densities is roughly the same. *Relative drag coefficient and relative diffusivity values were calculated by normalizing against that of water (there was no significant difference between the diffusivity values of the 2.5 mg/mL and 5.0 mg/mL groups).

	Water		2.5 mg/mL		5.0 mg/mL	
	Mean	Range	Mean	Range	Mean	Range
Drift velocity (m/s)	0.04	N/A	2.8*E* − 07	2.5*E* − 07 to 3.0*E* − 07	2.5*E* − 07	2.3*E* − 07 to 2.7*E* − 07
Accel. (m/s^2^)	0.0	N/A	−3.9*E* − 12	−4.4*E* − 12 to −3.3*E* − 12	−2.6*E* − 12	−2.8*E* − 12 to −2.2*E* − 12
Relative drag coef.*	1.0	N/A	5.9*E* + 04	−1.6*E* + 05 to 3.3*E* + 05	2.1*E* + 05	1.8*E* + 05 to 2.3*E* + 05
Relative diffusivity*	1.0	N/A	6.9*E* − 06	6.4*E* − 06 to 7.6*E* − 06	6.3*E* − 06	5.8*E* − 06 to 6.7*E* − 06
